# Involvement of APC and K-*ras* mutation in non-polypoid colorectal tumorigenesis

**DOI:** 10.1054/bjoc.1999.0869

**Published:** 1999-12-08

**Authors:** N Umetani, S Sasaki, T Masaki, T Watanabe, K Matsuda, T Muto

**Affiliations:** Department of Surgical Oncology, School of Medicine, The University of Tokyo, 7-3-1 Hongo, Bunkyo-ku, Tokyo 113-8655, Japan; First Department of Surgery, Kyorin University School of Medicine, 6-20-2 Shinkawa, Mitaka, Tokyo 181-8611, Japan; Cancer Institute Hospital, 1-37-1 Kami-Ikebukuro, Toshima-ku, Tokyo 170-8455, Japan

**Keywords:** APC, K- *ras*, tumorigenesis, colorectal carcinoma, adenoma-carcinoma sequence

## Abstract

The aim of this study was to clarify the role of APC and K- *ras* mutations in non-polypoid colorectal tumorigenesis. DNA from 63 adenomas (31 polypoid, 17 superficial elevated, 15 superficial depressed), 66 submucosally invasive carcinomas (47 polypoid, 19 non-polypoid) and 34 advanced carcinomas were examined for K- *ras* codon 12 point mutations and APC mutations in the mutation cluster region. K- *ras* mutation: the frequency in superficial depressed adenomas was lower than that in polypoid adenomas (0% vs 31%: *P* = 0.018). The frequency in non-polypoid carcinomas was lower than that in polypoid carcinomas (11% vs 56%: *P* = 0.0008), and was relatively low compared with that in polypoid adenomas (11% vs 31%). APC mutation: the frequency in superficial depressed adenomas was lower than that in polypoid adenomas (7% vs 43%: *P* = 0.016), and that in polypoid carcinomas was similar to that in non-polypoid carcinomas. Polypoid adenomas, polypoid carcinomas and advanced carcinomas had almost the same frequency. There may be some pathway other than the conventional adenoma-carcinoma sequence in development of non-polypoid carcinomas. The precursors of most non-polypoid carcinomas are considered to be de novo or superficial depressed adenomas. In this non-polypoid pathway, APC mutation seems to be requisite but K- *ras* mutation not. It is possible that new APC mutations are acquired after the development of superficial depressed adenomas. © Copyright 2000 Cancer Research Campaign


*British Journal of Cancer* (2000) **82**(1), 9–15
© 2000 Cancer Research Campaign
Article no. bjoc.1999.0869

### Involvement of APC and K-*ras* mutation in non-polypoid colorectal tumorigenesis

**N Umetani[1], S Sasaki[1], T Masaki[2], T Watanabe[1], K Matsuda[1] and T Muto[3]**

[1]Department of Surgical Oncology, School of Medicine, The University of Tokyo, 7-3-1 Hongo, Bunkyo-ku, Tokyo 113-8655, Japan; [2]First Department of Surgery, Kyorin University School of Medicine, 6-20-2 Shinkawa, Mitaka, Tokyo 181-8611, Japan; [3]Cancer Institute Hospital, 1-37-1 Kami-Ikebukuro, Toshima-ku, Tokyo 170-8455, Japan

**Summary** The aim of this study was to clarify the role of APC and K-*ras* mutations in non-polypoid colorectal tumorigenesis. DNA from 63 adenomas (31 polypoid, 17 superficial elevated, 15 superficial depressed), 66 submucosally invasive carcinomas (47 polypoid, 19 non-polypoid) and 34 advanced carcinomas were examined for K-*ras* codon 12 point mutations and APC mutations in the mutation cluster region. K-*ras* mutation: the frequency in superficial depressed adenomas was lower than that in polypoid adenomas (0% vs 31%: $P = 0.018$). The frequency in non-polypoid carcinomas was lower than that in polypoid carcinomas (11% vs 56%: $P = 0.0008$), and was relatively low compared with that in polypoid adenomas (11% vs 31%). APC mutation: the frequency in superficial depressed adenomas was lower than that in polypoid adenomas (7% vs 43%: $P = 0.016$), and that in polypoid carcinomas was similar to that in non-polypoid carcinomas. Polypoid adenomas, polypoid carcinomas and advanced carcinomas had almost the same frequency. There may be some pathway other than the conventional adenoma-carcinoma sequence in development of non-polypoid carcinomas. The precursors of most non-polypoid carcinomas are considered to be de novo or superficial depressed adenomas. In this non-polypoid pathway, APC mutation seems to be requisite but K-*ras* mutation not. It is possible that new APC mutations are acquired after the development of superficial depressed adenomas. © 2000 Cancer Research Campagin

**Keywords:** APC, K-*ras*; tumorigenesis; colorectal carcinoma; adenoma-carcinoma sequence

The adenoma–carcinoma sequence (Morson et al, 1968; Muto et al, 1975) in colorectal tumorigenesis has been widely accepted, and polypoid adenomas are recognized as the most likely precursor of colorectal carcinomas. A model of genetic alterations in the adenoma–carcinoma sequence has been proposed (Vogelstein et al, 1988). In this model, APC mutations occur at the initial step in adenoma formation (Powell et al, 1992), followed by point mutations of the K-*ras* gene, paralleling increases in adenoma size and grade of atypia, as well as mutations of various other tumour suppressor genes which accumulate during tumour development. APC mutations are found in more than 80% of sporadic colorectal adenomas and carcinomas (Kinzler et al, 1996), suggesting that APC acts as the 'gatekeeper' of colonic epithelial cell proliferation and that inactivation of this gene is required for net cellular proliferation. K-*ras* gene mutations have been found in nearly 50% of polypoid adenomas larger than 1 cm in diameter (Vogelstein et al, 1988).

Yamagata et al reported that the frequency of K-*ras* mutation in superficial adenomas was significantly lower than that in polypoid ademomas (Yamagata et al, 1994) and that non-polypoid type growth of submucosally invasive carcinomas (sm carcinomas) was associated with a lower frequency of K-*ras* mutation than the polypoid type (Yamagata et al, 1995), indicating carcinogenesis through superficial adenomas to be different from that occurring via polypoid adenomas with regard to genetic alterations. Therefore, it was suggested that there may be a pathway different from the conventional adenoma–carcinoma sequence. However, little is known about the role of APC gene mutations in this alternative pathway. Therefore, we investigated APC and K-*ras* mutations in colorectal tumours, focussing especially on the morphology and stage of these tumours.

*Received 22 March 1999*
*Revised 10 June 1999*
*Accepted 7 July 1999*

*Correspondence to:* N Umetani

### MATERIALS AND METHODS

### Tissues

Formalin-fixed, paraffin-embedded tissue samples of 63 colorectal adenomas and 100 invasive colorectal carcinomas, resected operatively or endoscopically from 1988 to 1998, were examined. All studied adenomas were classified as either mild atypia or moderate atypia. Tumours with high-grade atypia were excluded from this study to standardize the background. Cases with familial adenomatous polyposis (FAP) or hereditary non-polyposis colorectal cancer (HNPCC), meeting the criteria established by the International Collaborative Group on HNPCC (Vasen et al, 1991), were excluded.

### Morphological classification

Haematoxylin and eosin (H&E)-stained sections of each tumour were carefully examined by the same two pathologists (TM and KM) independently to confirm the diagnosis and morphological classification. Sixty-three adenomas were classified as 31 polypoid adenomas and 32 superficial adenomas, and the latter were

**Table 1** Morphological classification of examined tumours and sample number, co-existence of adenomatous component, K-*ras* codon 12 point mutation, and APC mutation in mutation cluster region

| | Depth[a] | Morphological classification | Number of tumours | Adenomatous component[b] | K-*ras*[c] | APC[d] |
|---|---|---|---|---|---|---|
| Adenoma | | Polypoid | 31 | – | 9/29 (31%)[f] | 13/30 (43%)[h] |
| | | Superficial elevated | 17 | – | 5/17 (29%) | 3/16 (19%) |
| | | Superficial depressed | 15 | – | 0/15 (0%) | 1/15 (7%) |
| Carcinoma | sm | Polypoid | 47 | 49%[e] | 24/43 (56%)[g] | 20/47 (43%) |
| | | Non-polypoid | 19 | 0% | 2/19 (11%) | 6/17 (35%) |
| | mp | | 34 | | 18/30 (60%) | 14/33 (42%) |
| Total | | | 163 | | | |

[a]sm: carcinomas invading into the submucosal layer; mp: carcinomas invading into the muscularis propria. [b]Co-existence ratio of adenomatous component. [c]Frequency of K-*ras* codon 12 mutation. [d]Frequency of APC mutation in mutation cluster region. [e]$P < 0.0001$, polypoid sm carcinoma vs. non-polypoid sm carcinoma by Fisher's exact test. [f]$P = 0.018$, polypoid adenoma vs. superficial depressed adenoma by Fisher's exact test. [g]$P = 0.0008$, polypoid sm carcinoma vs. non-polypoid sm carcinoma by Fisher's exact test. [h]$P = 0.016$, polypoid adenoma vs. superficial depressed adenoma by Fisher's exact test.

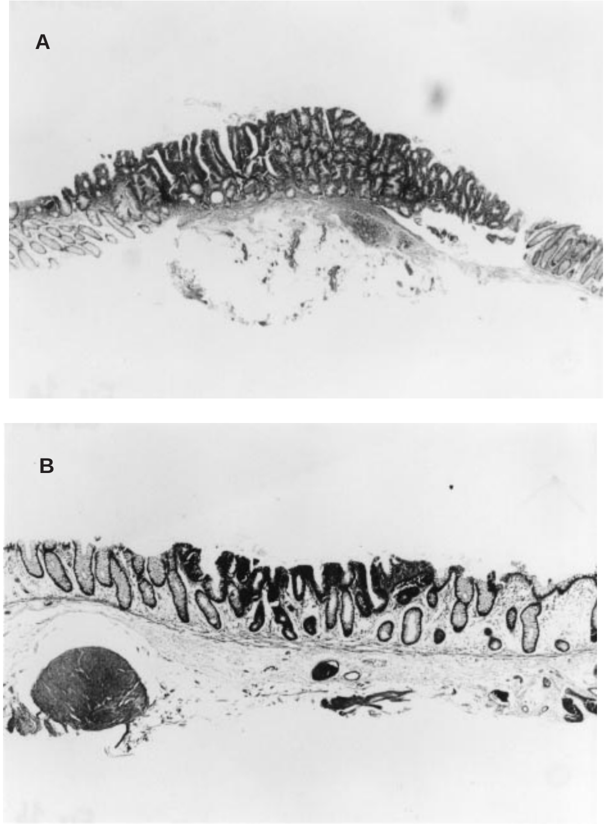

**Figure 1** Typical histologic appearances of a superficial elevated adenoma and a superficial depressed adenoma. A superficial adenoma is a lesion whose height above the muscularis mucosa is less than twice that of the surrounding normal mucosa. A superficial depressed adenoma is a lesion whose height above the muscularis mucosa is less than that of the surrounding normal mucosa in more than 50% of tumour basal area and the remainder are superficial elevated adenomas. **(A)** A typical superficial elevated adenoma. **(B)** A typical superficial depressed adenoma.

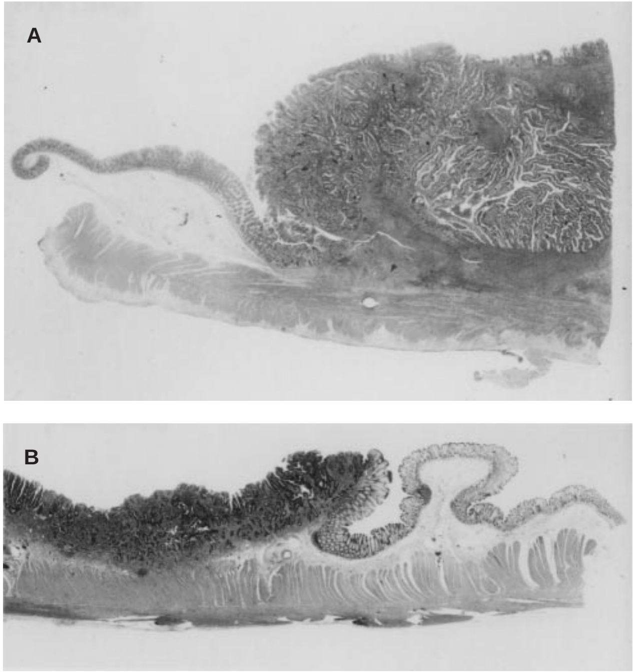

**Figure 2** Typical histologic appearances of a polypoid carcinoma and a non-polypoid carcinoma. A polypoid carcinoma grows with a polypoid margin overhanging the surrounding normal mucosa. A non-polypoid carcinoma has central ulceration and a non-polypoid margin, encircled by normal mucosa at the periphery. **(A)** A typical polypoid carcinoma. **(B)** A typical non-polypoid carcinoma subclassified as 17 superficial elevated adenomas and 15 superficial depressed adenomas according to their morphological features (Japanese Research Society for Cancer of the Colon and Rectum, 1983), as described below. A superficial adenoma is a lesion whose height above the muscularis mucosa is less than twice that of the surrounding normal mucosa. A superficial depressed adenoma is a lesion whose height above the muscularis mucosa is less than that of the surrounding normal mucosa in more than 50% of tumour basal area, and the remainder were superficial elevated adenomas. Typical histologic appearances of superficial adenomas are shown in Figure 1. Sixty-six sm carcinomas were classified as 47 polypoid carcinomas and 19 non-polypoid carcinomas according to their morphological features, as described below. A polypoid carcinoma grows with a polypoid margin overhanging the surrounding normal mucosa. A non-polypoid carcinoma has central ulceration and a non-polypoid margin, encircled by normal mucosa at the periphery. Typical histologic appearances are shown in Figure 2. Six specimens of sm carcinomas that could not be definitively classified were excluded from this study. Thirty-four

© 2000 Cancer Research Campaign

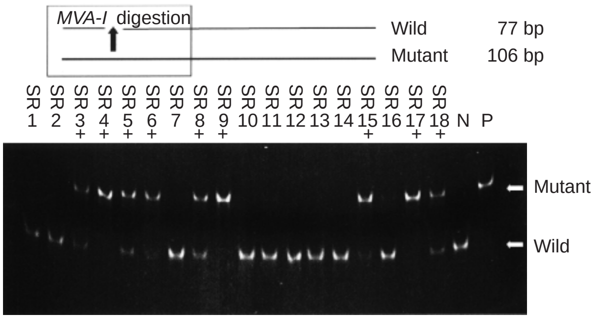

N: Normal control

P: Positive control

**Figure 3** Detection of K-*ras* codon 12 point mutations by two-step PCR-RFLP method. N: Normal control. P: Positive control. The arrow indicates the MVA-I digestion site in the second PCR product of wild-type allele, dividing the 106 base pair product into two fragments of 77 and 29 base pairs advanced carcinomas showed invasion into the muscularis propria (mp carcinomas) (Table 1). Advanced carcinomas were not subclassified.

### DNA extraction

Several 20-μm sections were obtained from the paraffin-embedded blocks, and neoplastic lesions were precisely dissected under microscopy with reference to the adjacent H&E-stained section. Contamination of normal tissue was limited to interstitial cells, and most normal epithelial cells were excluded from the specimens. DNA was extracted from each specimen after deparaffinization by treatment with sodium dodecyl sulphate (SDS)-proteinase K and phenol–chloroform–isoamyl alcohol as described previously (Goeltz et al, 1985). The DNA concentration was adjusted to 20 ng $\mu l^{-1}$.

### Detection of K-*ras* codon 12 point mutations

K-*ras* mutations were examined focusing on codon 12, because K-*ras* mutations in sporadic colorectal tumours occur predominantly (77–82%) at this codon (Bos et al, 1987; Oudejans et al, 1991). The DNA was amplified and analysed by two-step polymerase chain reaction (PCR)-restriction fragment length polymorphism (RFLP) according to a previously described method (Figure 3) (Levi et al, 1991; Yamagata et al, 1994). Negative and positive controls (wild-type DNA and mutated DNA) were run with each analysis. Since this method had enough high sensitivity, contamination of normal cells did not influence the result.

### Detection of APC MCR mutations

The APC area searched for mutations was restricted to the mutation cluster region (MCR) in which more than 80% of sporadic colorectal carcinomas harbour mutations (Miyoshi et al, 1992; Be'roud et al, 1996). We searched for APC mutations by PCR-single strand conformation polymorphism (SSCP) and direct sequencing methods. Five sets of overlapping PCR primers were synthesized to amplify exon 15 codons 1251–1536 including the whole MCR. The lengths of the five amplified fragments were in the range of 194–227 bases.

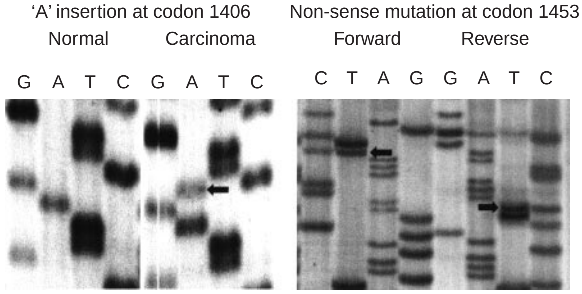

**Figure 4** Detection of mutations in APC MCR using direct sequencing method. **(A)** Arrowhead indicates 'A' insertion at codon 1406. **(B)** Arrowhead indicates 'C' to 'T' nucleotide change (Glu to Stop) at codon 1453, resulting in truncated protein

The primers used for PCR were as follows: MCR1F (forward): 5′-AAGGCTGCCACTTGCAAAG-3′; MCR1R (reverse): 5′-CTTCAGCTGACCTAGTTCC-3′; MCR2F (forward): 5′-GCTAATACCCTGCAAATAGC-3′; MCR2R (reverse): 5′-GGTGTCTGAGCACCACTTT-3′; MCR3F (forward): 5′-TTCTTCAGGAGCGAAATCTC-3′; MCR3R (reverse): 5′-GGGCTATCTGGAAGATCAC-3′; MCR4F (forward): 5′-CAGTGGAATGGTAAGTGGC-3′; MCR4R (reverse): 5′-AGCATCTGGAAGAACCTGG-3′; MCR5F (forward): 5′-GAGTGGACCTAAGCAAGCT-3′; MCR5R (reverse): 5′-TCCTGAACTGGAGGCATTAT-3′.

We screened all fragments for mutations by SSCP, and then determined the sequences of fragments that showed aberrant bands by the direct sequencing method. In SSCP, the DNA was amplified using a thermal cycler (Perkin-Elmer PCR9600) in 5-μl reaction mixtures containing 20-ng extracted tissue DNA, 0.1 pM each set of primers labelled with $^{32}P$-ATP, 200 μM deoxyribonucleoside triphosphates, 0.125 unit *Taq* polymerase (Perkin-Elmer(R) AmpliTaqGold), and a 10% volume of attached buffer according to the following protocol: 10 min at 95°C for polymerase activation, 40 cycles at 94°C for 30 s, 55°C for 2 min, 72°C for 1 min, followed by an additional 3 min at 72°C. The PCR products were electrophoresed on 5% acrylamide gels containing 6% glycerol at 4°C for 3 h at 1600 volts. The gels were dried and exposed to X-ray film. The fragments that showed aberrant migration patterns on SSCP were directly sequenced employing an Amersham Sequenase Version 2.0 7-deaza-dGTP kit with $^{35}S$-dCTP, following the manufacturer's protocol with the internal primers described below.

The internal sequencing primers used for direct sequencing were as follows: M CR1intF (forward): 5′-GCCACTTG-CAAAGTTTCTTCT-3′; MCR1intR (reverse): 5′-CAGCTGACC-TAGTTCCAATC-3′; MCR2intF (forward): 5′-TACCCTG-CAAAT AGCAGAAATA-3′; MCR2intR (reverse): 5′-TCTGAG-CACCACTTTTGGAG-3′; M CR3intF (forward): 5′-AGGAGC-GAAATCTCCCTCC-3′; MCR3intR (reverse): 5′-T ATCTG-GAAGATCACTGGGG-3′; MCR4intF (forward): 5′-GGAATG-GTAAGTGG CATTATAA-3′; MCR4intR (reverse): 5′-CTGGA-AGAACCTGGACCCT-3′; MCR5i ntF (forward): 5′-GACC-TAAGCAAGCTGCAGTA-3′; MCR5intR (reverse): 5′-GA ACTG-GAGGCATTATTCTTAA-3′.

Single-stranded template DNA for sequencing was prepared by asymmetric PCR (Gyllensten et al, 1988) with some modifications. All of the mutations detected were confirmed in the other direction and by performing the experiments at least twice

© 2000 Cancer Research Campaign 

**Table 2** APC somatic mutations in exon 15 codons 1251 to 1536 including MCR

| Case | Histology | Size (mm) | Depth[b] | Morphological classification | Codon | Nucleotide change | Mutation effect |
|---|---|---|---|---|---|---|---|
| Adenomas | | | | | | | |
| A2 | Mild | 25 | | Polypoid | 1286 | G insertion | Frame-shift |
| A7 | Mild | 18 | | Polypoid | 1303 | TT deletion | Frame-shift |
| A9 | Mild | 13 | | Polypoid | 1408 | CC deletion | Frame-shift |
| A12 | Mod. | 5 | | Polypoid | 1282 | C to G (ser to Stop) | Non-sense |
| A13 | Mild | 7 | | Polypoid | 1340 | C deletion | Frame-shift |
| A22 | Mild | 7 | | Polypoid | 1384 | CA deletion | Frame-shift |
| A29 | Mild | 8 | | Polypoid | 1313 | C insertion | Frame-shift |
| AH2 | Mild | 19 | | Polypoid | 1451 | A deletion | Frame-shift |
| AH17 | Mild | 12 | | Polypoid | 1376 | C deletion | Frame-shift |
| AH21 | Mod. | 6 | | Polypoid | 1315 | GAAAA deletion | Frame-shift |
| AH22 | Mild | 6 | | Polypoid | 1378 | G insertion | Frame-shift |
| AH23 | Mild | 9 | | Polypoid | 1362 | T deletion | Frame-shift |
| AH28 | Mild | 7 | | Polypoid | 1379 | C to A (Pro to Thr) | Amino acid change |
| FA7 | Mild | 2 | | Superficial Elevated | 1384 | CA deletion | Frame-shift |
| FA10 | Mild | 4 | | Superficial Elevated | 1394 | T deletion | Frame-shift |
| FA18 | Mild to mod. | 5 | | Superficial Elevated | 1414 | G to A (Gly to Gln) | Amino acid change |
| FA15 | Mild | 4 | | Superficial Depressed | 1464 | G insertion | Frame-shift |
| Invasive carcinomas | | | | | | | |
| SO4 | Well | 14 | sm | Polypoid | 1417 | A insertion | Frame-shift |
| SO6 | Well | 20 | sm | Polypoid | 1359 | A to T(Glu to Val) | Amino acid change |
| SO8 | Well | 5 | sm | Polypoid | 1415 | A insertion | Frame-shift |
| SO17 | Well | 17 | sm | Polypoid | 1406 | A insertion | Frame-shift |
| SR4 | Well | 20 | sm | Polypoid | 1497 | T insertion | Frame-shift |
| SR7 | Well | 9 | sm | Polypoid | 1470 | G to T (Glu to Stop) | Non-sense |
| SR9 | Well | 13 | sm | Polypoid | 1470 | G to T (Glu to Stop) | Non-sense |
| SR13 | Well | 20 | sm | Polypoid | 1309 | C to T (Gln to Stop) | Non-sense |
| SR17 | Well | 83 | sm | Polypoid | 1470 | G to T(Glu to Stop) | Non-sense |
| SE2 | Well | 13 | sm | Polypoid | 1384 | G deletion | Frame-shift |
| SE6 | Well | 12 | sm | Polypoid | 1373 | T insertion | Frame-shift |
| SE8 | Well | 15 | sm | Polypoid | 1407 | T deletion | Frame-shift |
| SE10 | Well | 9 | sm | Polypoid | 1275 | TA deletion | Frame-shift |
| SE11 | Well | 8 | sm | Polypoid | 1397 | A insertion | Frame-shift |
| SE15 | Well | 5 | sm | Polypoid | 1295 | A deletion | Frame-shift |
| ST11 | Well | 10 | sm | Polypoid | 1317 | TGGAA deletion | Frame-shift |
| ST16 | Well | 30 | sm | Polypoid | 1424 | AA deletion | Frame-shift |
| ST22 | Well | 30 | sm | Polypoid | 1309 | C to T (Gln to Stop) | Non-sense |
| ST34 | Well | 12 | sm | Polypoid | 1427 | A deletion | Frame-shift |
| ST35 | Mod. | 18 | sm | Polypoid | 1297 | GCAGAA deletion | Frame-shift |
| SR12 | Well | 3 | sm | non-Polypoid | 1327 | CGAAG deletion | Frame-shift |
| SO43 | Well | 7 | sm | non-Polypoid | 1422 | T insertion | Frame-shift |
| SO5 | Well | 14 | sm | non-Polypoid | 1542 | G insertion | Frame-shift |
| SE9 | Well | 9 | sm | non-Polypoid | 1399 | T to C (Leu to Pro) | Amino acid change |
| ST5 | Well | 20 | sm | non-Polypoid | 1314 | C insertion | Frame-shift |
| P1 | Well | 27 | mp | | 1453 | C to T (Glu to Stop) | Non-sense |
| P8 | Mod. | 45 | mp | | 1515 | T insertion | Frame-shift |
| P9 | Mod | 24 | mp | | 1416 | G to A (Glu to Lys) | Amino acid change |
| P11 | Well | 30 | mp | | 1374 | A insertion | Frame-shift |
| P12 | Muc. | 17 | mp | | 1329 | A insertion | Frame-shift |
| P13 | Por. | 50 | mp | | 1420 | A deletion | Frame-shift |
| P15 | Well | 24 | mp | | 1375 | C deletion | Frame-shift |
| P17 | Well | 45 | mp | | 1395 | T deletion | Frame-shift |
| P18 | Well | 32 | mp | | 1318 | GAACT deletion | Frame-shift |
| P19 | Well | 43 | mp | | 1307 | C deletion | Frame-shift |
| P20 | Well | 23 | mp | | 1328 | G insertion | Frame-shift |
| P21 | Well | 18 | mp | | 1385 | TA deletion | Frame-shift |
| P44 | Well | 34 | mp | | 1340 | CC deletion | Frame-shift |
| P47 | Well | 21 | mp | | 1493 | TTTA deletion | Frame-shift |

[a]For adenomas: mild: mild atypia; mod.: moderate atypia. For carcinomas: well: well differentiated adenocarcinoma; mod.: moderately differentiated adenocarcinoma; por.: poorly differentiated adenocarcinoma; muc: mucinous adenocarcinoma. [b]sm: carcinomas invading into the submucosal layer; mp: carcinomas invading into the muscularis propria.

*© 2000 Cancer Research Campaign*

**Table 3** Correlation between mutations of APC MCR and K-*ras* codon 12

| | Morphological classification | K-*ras*[a] | APC[b] + | APC[b] – | |
|---|---|---|---|---|---|
| Adenomas | Polypoid | + | 2 | 5 | |
| | | – | 9 | 11 | NS[c] |
| | Superficial elevated | + | 1 | 4 | |
| | | – | 2 | 9 | NS |
| | Superficial depressed | + | 0 | 0 | |
| | | – | 1 | 13 | NS |
| | (Total) | + | 3 | 9 | |
| | | – | 12 | 33 | NS |
| sm ca. | Polypoid | + | 12 | 12 | |
| | | – | 6 | 12 | NS |
| | non-Polypoid | + | 0 | 2 | |
| | | – | 5 | 8 | NS |
| | (Total) | + | 12 | 14 | |
| | | – | 11 | 20 | NS |
| mp ca. | | + | 6 | 11 | |
| | | – | 5 | 7 | NS |

[a]K-*ras* codon 12 mutation; +: positive for mutation; –: negative for mutation. [b]APC mutation in mutation cluster region; +: positive for mutation; –: negative for mutation. [c]NS: not significant.

(Figure 4). To distinguish and exclude mutations resulting in amino acid replacements from polymorphism, we determined the sequence of DNA obtained from normal tissue of the same patient (data not shown).

### Statistical analysis

All of the *P*-values were calculated by Fisher's exact test. According to Bonferroni's inequality, *P*-values smaller than 0.025 are statistically significant on multiple comparisons.

### RESULTS

### Tumour morphology and histology

Most of the sm carcinomas were well differentiated carcinomas (52/61, 85%), and the remaining ones were moderately differentiated carcinomas. The percentage of each histological type was identical between polypoid carcinomas and non-polypoid carcinomas (polypoid: well 87%, mod, 13%; non-polypoid: well 84%, mod, 16%). As shown in Table 1, 49% of polypoid carcinomas contained an adenomatous component, however, none of non-polypoid carcinomas did. There was a statistically significant difference in the frequency of adenomatous components between polypoid carcinomas and non-polypoid carcinomas ($P < 0.0001$).

### K-*ras* mutations

As shown in Table 1, K-*ras* mutations at codon 12 were present in nine of 29 (31%) polypoid adenomas, five of 17 (29%) superficial elevated adenomas and none of 15 (0%) superficial depressed adenomas. The frequency of mutation in superficial depressed adenomas was significantly lower than that in polypoid adenomas (0% vs 31%: $P = 0.018$). The frequency of mutation in non-polypoid carcinomas was significantly lower than that in polypoid carcinomas (11% vs 56%: $P = 0.0008$), and was relatively low compared with that in polypoid adenomas (11% vs 31%).

### APC mutations

Among 56 mutations detected in APC MCR, 51 (91%) were frame-shift or non-sense mutations resulting in truncated proteins, and the remaining five (9%) were one base replacements resulting in single amino acid changes (Table 2). As shown in Table 1, APC mutations were found in 13 of 30 (43%) polypoid adenomas, three of 16 (19%) superficial elevated adenomas, one of 15 (7%) superficial depressed adenomas, 20 of 47 (43%) polypoid carcinomas, six of 17 (35%) non-polypoid carcinomas and 14 of 33 (42%) advanced carcinomas. Polypoid adenomas, polypoid carcinomas and advanced carcinomas showed almost the same frequency of APC mutation. The frequency of APC mutation in superficial depressed adenomas was significantly lower than that in polypoid adenomas (7% vs 43%: $P = 0.016$). The frequency of APC mutation in polypoid carcinomas was similar to that in non-polypoid carcinomas, with no significant difference between them.

### Relation between APC and K-*ras* mutations

As shown in Table 3, no correlation was observed between APC mutations and K-*ras* mutations at any tumour stage.

### DISCUSSION

To investigate the involvement of APC and K-*ras* mutations in colorectal tumorigenesis, we collected colorectal tumours from every morphological classification at every stage in the pathway. We could collect only 15 superficial depressed adenomas because they were very rare and it was rather difficult to find them. Other types of adenomas and carcinomas were collected randomly within the same period. The proportion of superficial depressed adenomas examined in this study was higher than that expected from the natural distribution.

Shimoda et al have conducted a pioneering study focusing on the morphology of early colorectal carcinomas, in which these carcinomas were classified into polypoid growth carcinomas, based on intramucosal proliferation of adenoma and carcinoma, and non-polypoid growth carcinomas without intramucosal protuberant growth (Shimoda et al, 1989).

We classified sm carcinomas into polypoid carcinomas and non-polypoid carcinomas. This classification is relevant and easily applicable to all early colorectal carcinomas including mucosal carcinomas, because only peripheral overhanging of the tumour is taken into consideration. This classification seems somewhat subjective, but most tumours (66 out of 72) were definitely classified with no discrepancy. In this study, the significant difference between polypoid carcinomas and non-polypoid carcinomas in the frequency of existence of adenomatous components strongly supports that this classification is suitable for the investigation of colorectal tumorigenesis. There was no correlation between morphological and histological classification. In consideration of morphology, it can be assumed that polypoid carcinomas mainly developed from polypoid adenomas through the conventional adenoma–carcinoma sequence and that non-polypoid carcinomas developed de novo or from superficial depressed adenomas without demonstrating any substantial morphological changes. Superficial elevated adenomas seemed to be intermediate lesions of polypoid adenomas and superficial depressed adenomas.

As Vogelstein et al suggested, K-*ras* mutation is assumed to be an early event in the adenoma–carcinoma sequence, contributing

© 2000 Cancer Research Campaign to growth and dysplastic change in adenoma development (Vogelstein et al, 1988). However, they did not pay consideration to superficial adenomas or special types of carcinoma such as non-polypoid carcinoma. Therefore, we designed this study and found that the frequency of K-*ras* mutation in non-polypoid carcinomas was significantly lower than that in polypoid carcinomas, and that it was lower than that in polypoid adenomas. Furthermore, we demonstrated that the frequency of K-*ras* mutation in superficial depressed adenomas was significantly lower than that in polypoid adenomas. These results support the possibility that the precursors of non-polypoid carcinomas are not polypoid adenomas, but are de novo or superficial depressed adenomas, and non-polypoid carcinomas do not progress through the conventional adenoma–carcinoma sequence. This hypothesis fits the assumption that polypoid adenomas do not progress into non-polypoid carcinomas without bold morphological changes. K-*ras* mutations do not appear to be requisite for the development of these tumours.

In this study, we also searched for APC mutations and demonstrated that polypoid adenomas, polypoid carcinomas and advanced carcinomas have almost the same frequency and distribution pattern of APC mutations. This result is compatible with the concept that APC acts as the ‘gatekeeper’ of colonic epithelial cell proliferation. The frequency of APC mutation in our study is apparently lower than those reported previously (Kinzler et al, 1996). This may be because we searched for mutations only in MCR, and the SSCP method used for screening has limited detection capability. This limitation was mainly due to the quantity and quality of obtained DNA. Furthermore, we demonstrated for the first time that the frequency of APC mutation in polypoid carcinomas was similar to that in non-polypoid carcinomas, which indicates that the APC mutation is requisite for carcinomatous development through the non-polypoid pathway. Supposing that superficial depressed adenomas develop into non-polypoid carcinomas, the finding of a low frequency of APC mutation in superficial depressed adenomas supports the hypothesis that new APC mutations are acquired during carcinomatous change. To confirm that this hypothesis is correct, accumulation of clinical observations and further investigation are required. Additionally, the authors found no correlation between APC mutations in MCR and K-*ras* mutations at codon 12 at any tumour stage, raising the possibility that APC and K-*ras* gene mutations occur independently in colorectal tumorigenesis.

In this study, we could not exclude the possibility that the difference of tumour size in each classification influenced the result. However, since the difference of tumour size is an essential element determining the morphological type, and the objective of the present study is based on this, we consider that it is impossible to exclude the influence of tumour size completely.

In this study the mutation of APC might have been masked by DNA from contaminated normal cells, and therefore the frequency of mutation might have been evaluated to be as falsely low. But the quantity of contaminated normal DNA was considered to be not enough to mask the mutations, and was assumed to be constant in each classification, because we precisely cut out the neoplastic lesions by the microdissection method and eliminated contamination with normal cells. Therefore we considered that the influence on the result would be averaged and would thus be negligible.

There was no somatic APC mutations at two of the most common sites: 5 bp deletion at codon 1309, and non-sense mutation at codon 1450 (Miyaki et al, 1995), but we could not find any explicable reason.

Because the number of investigated superficial depressed adenomas was small, it is necessary to collect more specimens to obtain a concrete conclusion. To confirm the different mechanism between the conventional adenoma–carcinoma sequence and non-polypoid pathway, we are conducting further studies focussing on the clinical and genetic characteristics of superficial colorectal lesions.

In summary, we conclude that there may be some pathway other than the conventional adenoma–carcinoma sequence in development of non-polypoid carcinomas. The precursors of most non-polypoid carcinomas are considered to be not polypoid adenomas, but de novo or superficial depressed adenomas. For carcinomatous development through this non-polypoid pathway, APC mutation seems to be requisite, but K-*ras* mutation not. It is possible that new APC mutations are acquired after the development of superficial depressed adenomas.

### ACKNOWLEDGEMENTS

The authors are grateful to the surgeons and other physicians at Kitazato University Hospital, Tokyo, Fukuoka University Chikushi Hospital, Fukuoka, Kishiwada Tokushukai Hospital, Osaka and Sanraku Hospital, Tokyo, for kindly providing tissue samples. This study was supported by a Grant-in-aid from the Ministry of Health and Welfare.

### REFERENCES

Be’roud C and Soussi T (1996) APC gene: database of germline and somatic mutations in human tumors and cell lines. *Nucleic Acids Res* **24**: 121–124

Bos JL, Fearon ER, Hamilton SR, Verlaan-de Vries M, Van Boom JH, Van der Eb AJ and Vogelstein B (1987) Prevalence of ras gene mutations in human colorectal cancers. *Nature* **327**: 293–297

Goeltz SE, Hamilton SR and Vogelstein B (1985) Purification of DNA from formaldehyde fixed and paraffin embedded human tissue. *Biochem Biophys Res Commun* **130**: 118–126

Gyllensten UB and Erlich HA (1988) Generation of single-stranded DNA by the polymerase chain reaction and its application to direct sequencing of the HLA-DQA locus. *Proc Natl Acad Sci USA* **85**: 7652–7656

Japanese Research Society for Cancer of the Colon and Rectum (1983) General rules for clinical and pathological studies on the cancer of the colon, rectum and anus. Part II. Histopathological classification. *Jpn J Surg* **13**: 574–598

Kinzler KW and Vogelstein B (1996) Lessons from hereditary colorectal cancer. *Cell* **87**: 159–170

Levi S, Urbano-Ispizua A, Gill R, Thomas DM, Gilbertson J, Foster C and Marshall CJ (1991) Multiple K-*ras* codon 12 mutations in cholangiocarcinomas demonstrated with a sensitive polymerase chain reaction technique. *Cancer Res* **51**: 3497–3502

Miyaki M, Tanaka K, Kikuchi-Yanoshita R, Muraoka M and Konishi M (1995) Familial polyposis: recent advances. *Crit Rev Oncol Hematol* **19**: 1–31

Miyoshi Y, Nagase H, Ando H, Horii A, Ichii S, Nakatsuru S, Aoki T, Miki Y, Mori T and Nakamura Y (1992) Somatic mutations of the APC gene in colorectal tumors: mutation cluster region in the APC gene. *Hum Mol Genet* **1**: 229–233

Morson BC (1968) Precancerous and early malignant lesions of the large intestine. *Br J Surg* **55**: 725–731

Muto T, Bussey HJR and Morson BC (1975) The evolution of cancer of the colon and rectum. *Cancer* **36**: 2251–2270

Oudejans JJ, Slebos RJC, Zoetmulder FAN, Mooi WJ and Rodenhuis S (1991) Differential activation of ras genes by point mutation in human colon cancer with metastases to either lung or liver. *Int J Cancer* **49**: 875–879

Powell SM, Zilz N, Beazer-Barclay Y, Bryan TM, Hamilton SR, Thibodeau SN, Vogelstein B and Kinzler KW (1992) APC mutations occur early during colorectal tumorigenesis. *Nature* **359**: 235–237

Shimoda T, Ikegami M, Fujisaki J, Matsui T, Aizawa S and Ishikawa E (1989) Early colorectal carcinoma with special reference to its development de novo. *Cancer* **64**: 1138–1146

© 2000 Cancer Research Campaign

Vasen HFA, Mecklin JP, Khan PM and Lynch HT (1991) The International Collaborative Group on Hereditary Non-Polyposis Colorectal Cancer (ICG-HNPCC). *Dis Colon Rectum* **34**: 424–425

Vogelstein B, Fearon ER, Hamilton SR, Kern SE, Preisinger AC, Leppert M, Nakamura Y, White R, Smits AM and Bos JL (1988) Genetic alterations during colorectal-tumor development. *N Engl J Med* **319**: 525–532

Yamagata S, Muto T, Uchida Y, Masaki T, Sawada T, Tsuno N and Hirooka T (1994) Lower incidence of K-*ras* codon 12 mutation in flat colorectal adenomas than in polypoid adenomas. *Jap J of Cancer Res* **85**: 147–151

Yamagata S, Muto T, Uchida Y, Masaki T, Higuchi Y, Sawada T and Hirooka T (1995) Polypoid growth and K-*ras* codon 12 mutation in colorectal cancer. *Cancer* **75**: 953–957

*© 2000 Cancer Research Campaign*
